# Measurement over 1 Year of Neutralizing Antibodies in Cattle Immunized with Trivalent Vaccines Recombinant Alpha, Beta and Epsilon of *Clostridium perfringens*

**DOI:** 10.3390/toxins13090594

**Published:** 2021-08-26

**Authors:** Cleideanny C. Galvão, José D. Barbosa, Carlos M. C. Oliveira, Denis Y. Otaka, Paulo R. O. Silva, Marcos R. A. Ferreira, Clóvis Moreira Júnior, Fabricio R. Conceição, Felipe M. Salvarani

**Affiliations:** 1 Instituto de Medicina Veterinária, Universidade Federal do Pará, Castanhal, Pará CEP 68740-970, Brazil; annymedvet@gmail.com (C.C.G.); diomedes@ufpa.br (J.D.B.); cmagno@ufpa.br (C.M.C.O.); otaka@veterinario.med.br (D.Y.O.); irodrigoliveira@icloud.com (P.R.O.S.); 2 Centro de Desenvolvimento Tecnológico, Núcleo de Biotecnologia, Universidade Federal de Pelotas, Pelotas, Rio Grande do Sul CEP 96160-000, Brazil; marcosferreiravet@gmail.com (M.R.A.F.); clovismoreirajr@live.com (C.M.J.); fabricio.rochedo@ufpel.edu.br (F.R.C.)

**Keywords:** immunology, biotechnology, antibody curve, recombinant alpha protein, recombinant beta protein, recombinant epsilon protein, serum neutralization, vaccine protocols

## Abstract

The alpha (CPA), beta (CPB) and epsilon (ETX) toxins of *Clostridium perfringens* are responsible for causing diseases that are difficult to eradicate and have lethal potential in production animals. Vaccination of herds is still the best control strategy. Recombinant clostridial vaccines have shown good success at inducing neutralizing antibody titers and appear to be a viable alternative to the conventional production of commercial clostridial toxoids. Research is still needed on the longevity of the humoral immune response induced by recombinant proteins in immunized animals, preferably in target species. The objective of this study was to measure the humoral immune response of cattle immunized with trivalent vaccines containing the recombinant proteins alpha (rCPA), beta (rCPB) and epsilon (rETX) of *C. perfringens* produced in *Escherichia coli* at three different concentrations (100, 200, and 400 µg) of each protein for 12 months. The recombinant vaccines containing 200 (RV2) and 400 µg (RV3) yielded statistically similar results at 56 days. They performed better throughout the study period because they induced higher neutralizing antibody titers and were detectable for up to 150 and 180 days, respectively. Regarding industrial-scale production, RV2 would be the most economical and viable formulation as it achieved results similar to RV3 at half the concentration of recombinant proteins in its formulation. However, none of the vaccines tested induced the production of detectable antibody titers on day 365 of the experiment, the time of revaccination typically recommended in vaccination protocols. Thus, reiterating the need for research in the field of vaccinology to achieve greater longevity of the humoral immune response against these clostridial toxins in animals, in addition to the need to discuss the vaccine schedules and protocols adopted in cattle production.

## 1. Introduction

Brazil has a herd of more than 214 million cattle and produces approximately 10 million tons of meat for internal and external consumption, making it one of the main beef-producing and beef-exporting countries in the world [1]. Brazilian cattle production faces health challenges from diseases that are difficult to eradicate, such as clostridiosis, which are responsible for causing herd mortality, resulting in economic losses of approximately USD 350 million per year for the national agribusiness production sector [2,3,4].

*Clostridium perfringens* is an aerotolerant, Gram-positive, endospore-forming anaerobic bacillus that is ubiquitous and is commensal in the gastrointestinal tract of humans and other healthy animals [5,6]. This bacterium can produce an average of 20 toxins, which are currently classified into seven toxinotypes (A–G) based on the presence of six main toxins: alpha (CPA), beta (CPB), epsilon (ETX), iota (ITX), enterotoxin (CPE) and NetB [6,7,8,9]. They cause important myonecrotizing, neurological and enteric conditions in production animals (Table 1) [2,3]. The acute and lethal effects caused by these toxins hinder the treatment of affected animals and vaccination is still the most effective strategy for the control and prevention of these diseases [2,9].

The commercial clostridial vaccines available on the market are mostly polyvalent and are composed mainly of toxoids, which are formaldehyde-inactivated clostridial toxins [3,10]. However, the production of these conventional toxoids has disadvantages because it requires well-characterized and properly preserved stable strains, specific culture media, a controlled anaerobiotic environment and several inactivation and detoxification stages, making the industrial process laborious, in addition to requiring rigorous biosafety protocols [11,12]. In addition, variations in antigen concentrations may occur in commercial vaccines according to the different production processes [3,13,14], risk of incomplete toxoid inactivation and a possible residual toxicity for vaccinated animals [2].

Recombinant clostridial vaccines are viable alternatives to conventional commercial production [2,3,4,10,11,15,16,17,18,19,20], eliciting the production of neutralizing antibodies to within the levels required by law. However, studies on the modulation of the animal immune system are still needed to establish the longevity of the humoral immune response induced by different doses of recombinant proteins [3]. Thus, the objective of the present study was to investigate the 1-year dynamics of the neutralizing antibody titers of cattle immunized with trivalent vaccines containing the recombinant proteins alpha (rCPA), beta (rCPB) and epsilon (rETX) of *C. perfringens* produced in *Escherichia coli*.

## 2. Results

### 2.1. Safe Vaccine Formulations for Use in Cattle

The sterility test did not indicate fungal or bacterial growth in any of the three produced recombinant vaccine formulations during the 21 days of observation. In the safety test, the animals did not show any type of local or systemic reaction, indicating the absence of toxicity.

### 2.2. Antibody Vary According to the Vaccine Formulation

The neutralizing antibody titer varied according to vaccine formulation, as did the number of animals that presented and maintained each titer throughout the study. At 56 days, all vaccines had induced a humoral immune response, but RV2 and RV3 were the only vaccines to achieve 100% (8/8) seroconversion against the three toxins, while the commercial vaccine only reached 100% seroconversion against the ETX toxin (Table 2). The RV1 vaccine, containing 100 μg, had the lowest seroconversion percentages: 37.5% (3/8) against CPA and CPB, 62.5% (5/8) against ETX. The animals in the negative control group (G5) showed no detectable anti-CPA, anti-CPB or anti-ETX titer over the 12 months of the study and are not included in the comparison tables.

**Figure 1:**
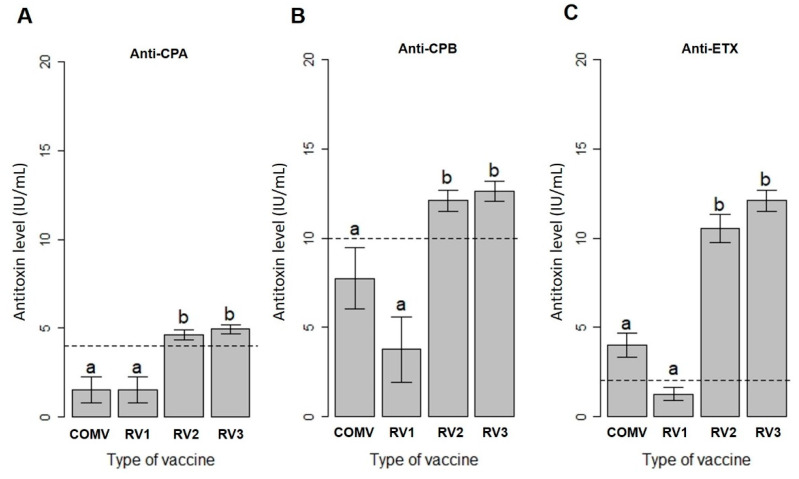
Analysis of the mean titers of alpha (anti-CPA), beta (anti-CPB) and epsilon antitoxins (anti-ETX) of *Clostridium perfringens* in cattle immunized with the commercial vaccine (COMV) or with the recombinant trivalent vaccines (RV1, RV2, RV3) on day 56 after the first vaccination. Lowercase letters (a,b) were used to indicate whether the groups were statistically equal or different when compared by Dunn’s post hoc test (*p* < 0.05). The dashed lines show the minimum level of neutralizing antibody titers against each toxin required by law (4, 10 and 2 IU/mL for alpha, beta and epsilon, respectively). (**A**) Mean titers of neutralizing antibodies against CPA, (**B**) mean titers of neutralizing antibodies against CPB, and (**C**) mean titers of neutralizing antibodies against ETX.

The Kruskal–Wallis test showed that vaccine type affected the production of anti-CPA (X^2^_(3)_ = 19.265; *p* < 0.0002), anti-CPB (X^2^_(3)_ = 19.544; *p* < 0.0002) and anti-ETX titers (X^2^_(3)_ = 26.347; *p* < 0.000000). The RV2 and RV3 vaccines were statistically similar in their induction of mean titers of antibodies against CPA, CPB and ETX, being the only formulations to induce the minimum levels against the three toxins required by law (4, 10 and 2 IU/mL, respectively). These two groups had significantly higher mean titers than the others. The *p*-values of the pairwise comparisons using Dunn’s post hoc test indicated that COMV differed from RV2 and RV3 in the induction of mean titers for anti-CPA (respectively, *p* < 0.02; *p* < 0.004), anti-CPB (*p* < 0.005; *p* < 0.006) and anti-ETX (*p* < 0.02; *p* < 0.01) at 56 days after immunization. COMV was statistically equal to RV1 against the three toxins analyzed and only reached the minimum required titer of antibodies against the ETX toxin, whereas RV1 did not reach the minimum level required by international law against any toxin (Figure 1).

### 2.3. The Amount of Recombinant Protein Influences the Longevity of the Humoral Immune Response

The mean titers of antibodies against CPA, CPB and ETX peaked at 56 days and remained detectable only until day 180 after the first vaccination. Starting on day 210, no experimental group showed the minimum antibody titer established by law against the CPA, CPB or ETX toxin of *C. perfringens* (4, 10, 2 IU/mL) by the seroneutralization technique. On day 56, only the recombinant formulations RV2 and RV3 achieved mean antibody titers higher than the minimum values. Both showed a similar behavior throughout the study, with higher antibody titers than the commercial vaccine and RV1. However, of all vaccines, only RV3 induced detectable neutralizing antibody titers through day 180.

**Figure 2:**
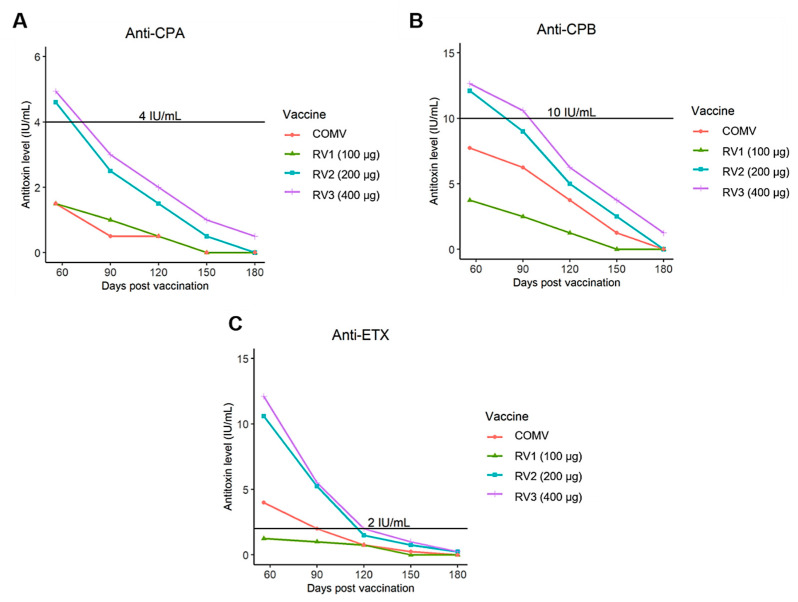
(**A**) Mean titers of *Clostridium perfringens* alpha antitoxin (anti-CPA) in cattle immunized with the commercial vaccine (COMV) and cattle given the recombinant trivalent vaccines (RV1, RV2 and RV3) on days 56, 90, 120, 150 and 180 after the first vaccination. (**B**) Mean titers of *Clostridium perfringens* beta antitoxin (anti-CPB) in cattle immunized with the commercial vaccine (COMV) or a recombinant trivalent vaccine (RV1, RV2 and RV3) on days 56, 90, 120, 150 and 180 after the first vaccination. (**C**) Mean titers of *Clostridium perfringens* epsilon antitoxin (anti-ETX) in cattle immunized with commercial vaccine (COMV) or a recombinant trivalent vaccine (RV1, RV2 and RV3) on days 56, 90, 120, 150 and 180 after the first vaccination.

The RV1 and COMV groups did not reach the minimum mean antibody values at nearly any time in the study period and presented longevity up to 120 and 150 days, respectively. Starting on day 90, all vaccine groups showed a reduction in antibody titers, but the mean anti-ETX values remained within those required by law for a longer time than anti-CPA and anti-CPB (Figure 2).

## 3. Discussion

Recombinant proteins have shown promising results in the induction of neutralizing antibodies in different species, reaching the minimum titers required by law and higher than those obtained with commercial vaccines [3,4,15,16,17,18,19,20,21]. However, most of these studies involve potency tests, evaluating the production of neutralizing antibodies only at 56 days after the first vaccination, only two of them involving vaccines against botulism [12,18] among those that have evaluated the duration of the induced protection time in immunized animals. This study is the first to measure the longevity of the humoral immune response of cattle immunized with trivalent vaccines containing the recombinant proteins alpha (rCPA), beta (rCPB) and epsilon (rETX) of *C. perfringens* at three different concentrations (100, 200, and 400 µg) of each protein for a period of 1 year.

The measured antibody titers varied according to the vaccine formulation and over time. In the animals vaccinated with the highest concentrations tested (200 and 400 µg), higher titers were induced than those required by law, and these were the only animals to achieve 100% (8/8) seroconversion for anti-CPA, anti-CPB and anti-ETX on day 56. RV2 and RV3 had statistically equal mean antibody titers at 56 days, with 4.62 and 4.94 IU/mL for anti-CPA, 12.1 and 12.6 IU/mL for anti-CPB and 10.6 and 12.1 IU/mL for anti-ETX, respectively. These were the formulations that maintained the highest mean antibody titers throughout the study. These results are similar to those described by Moreira et al. [12] in cattle and by Otaka et al. [18] in buffaloes, who measured the longevity of the immune response of these animals immunized with bivalent recombinant vaccines against botulinum neurotoxins (BoNTs) C and D over 365 days. Those authors used the same concentrations adopted in the present study (100, 200, 400 µg) and demonstrated that the concentrations of 200 and 400 µg also induced higher mean and longer lasting antibody titers than other formulations, including the commercial vaccine. This reinforces the idea that the levels of neutralizing antibody titers and the longevity of the immune response are directly related to the protein concentration per dose used in a vaccine.

In this study, the group of animals vaccinated with RV1 did not reach the minimum mean antitoxin titers for CPA, CPB or ETX on any of the evaluated days using the serum neutralization technique. The RV2 vaccine containing 200μg was the lowest recombinant protein concentration capable of inducing 100% seroconversion in the animals against the three toxins at 56 days after vaccination. It showed results statistically similar to RV3 in regard to the mean antibody titers throughout most of the study. Freitas et al. [19] tested, in horses, bivalent vaccines against *C. perfringens* rCPA and rCPB, obtained by the same cloning and expression technique used in the present study, at concentrations of 100, 200 and 400 µg per dose. At 56 days after immunization, the authors also found 200 µg as the lowest concentration capable of inducing the minimum antibody titers required by law against these toxins, demonstrating that the recombinant formulations produced using this technology show similar performance even in different species. These results indicate that the 100µg formulation may contain an inadequate concentration of antigens that can stimulate a satisfactory and lasting immune response against these toxins. However, they also indicate that 200 µg would be the minimum ideal concentration of recombinant proteins to induce an international law-compliant humoral immune response. In addition, RV2 showed results similar to RV3, and because it contains half the concentration of recombinant proteins in its formulation, it can be seen as a more economically viable option for large-scale industrial production.

The commercial vaccine had a significantly lower performance than RV2 and RV3 throughout the study. In addition, it induced antibody titers below the minimum values required by law against CPA and CPB in most animals, reaching a 100% seroconversion rate only against ETX at 56 days after vaccination. When compared to the recombinant formulations, COMV was statistically similar to only the weakest experimental vaccine (RV1). Regarding the longevity of the induced immune response, it showed detectable antibody levels up to 150 days, but only for anti-CPB and anti-ETX. In recent years, other studies have also reported that the antibody titers induced by commercial clostridial vaccines were lower than those obtained with recombinant vaccines using the same adjuvant, aluminum hydroxide [10,15,16,17,19]. Augusto de Oliveira et al. [22] evaluated the humoral immune response of cattle inoculated with polyvalent commercial vaccines containing *C. botulinum* toxoids type C and D (BoNTs) and the *C. perfringens* ETX toxoid for 1 year. During the study period, only 12.5% of the animals had minimum levels of neutralizing antibodies against all analyzed antigens, whereas the present study obtained 100% seroconversion for anti-CPA, anti-CPB and anti-ETX at 56 days with the RV2 and RV3 formulations. As a quality control of commercial clostridial vaccines, annual potency tests are performed on model species in which the neutralizing antibody titers are measured by the serum neutralization technique weeks after immunization of these animals, as described in MAPA norm 49 [23]. However, studies indicate a discrepancy in the titers of neutralizing antibodies induced in the target species when compared to those obtained in animal models used in official tests [3,4]. These data indicate that commercial clostridial vaccines, although approved through potency tests, have a low efficacy in the humoral immune response induced in target species, such as in cattle in the field. This reaffirms the importance of renewing the discussion about animal models that more accurately reflect the potency of these formulations for commercial use. In addition, recent studies have reported the immunodominance of some clostridial antigens present in commercial polyvalent vaccines after observing significant differences in the levels of neutralizing antibodies induced in sheep [24] and cattle [22] against each antigen analyzed, indicating possible antigenic competition. Although this competition mechanism is not fully elucidated for clostridial vaccines, studies have analyzed the factors underlying this phenomenon in sheep immunized with different types of vaccines against *Dichelobacter nodosus* and reported that this term has been used to describe the tendency of some polyvalent vaccines to induce lower immune responses than those achieved with vaccines containing only one component. They also highlighted that such competition may be related to the presence of many structurally related antigens in the formulations [25,26], another possible explanation for the low levels of protection obtained with commercial polyvalent clostridial vaccines.

All formulations tested in this study reached their maximum peak antibody levels at 56 days and they decreased starting on day 90 after the first vaccination. Compared with the anti-CPA and anti-CPB titers, the mean anti-ETX titers decreased more slowly and remained above the minimum values required for longer (120 days). Moreira et al. [3] tested the immunogenicity of this recombinant trivalent vaccine in sheep, goats and cattle, prepared with the same inputs and protocols as in the present study. They observed that rETX induced higher levels of neutralizing antibodies than rCPA and rCPB during the study, both in the potency test in rabbits and in the other evaluated species. This recombinant version of the *C. perfringens* ETX toxin has been shown to induce high levels of neutralizing antibodies, surpassing the titers obtained with commercial vaccines, either combined with other recombinant proteins, as in this study, or in experiments evaluating its performance alone in a no purified version [11]. The results described here indicate that rETX is a promising molecule for the production of a recombinant vaccine at the commercial level and that the differences found in the humoral immune response levels against each analyzed antigen are related to the hypothesis of antigenic competition that has been observed in commercial polyvalent clostridial vaccines [22,24] and different toxins produced by *Clostridium* species. Further related are experiments to compare the antibody titers induced by conventional and recombinant vaccines, mono- or polyvalent, to measure the influence of the number of vaccine antigens contained in the formulation on the level of protection generated in the animals and the duration of this response against each antigen.

Regarding the longevity of the induced immune response, although the RV2 and RV3 vaccines performed better in this study, they still did not induce detectable antibody titers for longer than 6 months after the first vaccination. Starting on day 210, it was no longer possible to measure antibody titers in any of the immunized cattle from any experimental group using the serum neutralization technique. It is noteworthy that the protocols and schedules for immunization against clostridiosis recommend revaccination only annually [27]. Thus, according to the observed results, the animals immunized with COMV would, in theory, remain unprotected for approximately 210 days, and those vaccinated with RV3 for 180 days, before the annual booster dose. This low longevity of the immune response was also observed by Moreira et al. [12] and Otaka et al. [18] with the use of recombinant proteins, as well as in the experiment by Augusto de Oliveira et al. [22] in which cattle immunized with commercial polyvalent clostridial vaccines showed measurable neutralizing antibody titers only up to 60 days for ETX, 120 days for BoNT C and 180 days for BoNT D, according to the serum neutralization technique. These data indicate that the short duration of the humoral immune response is a reality observed in animals immunized with commercial toxoids or with recombinant vaccines, demonstrating the importance of planning new clostridial vaccination protocols and schedules and proposing adjustments to existing ones. The addition of more doses at smaller intervals, such as biannual revaccination or adjustments in the intervals between the first and second doses of the initial vaccination protocol, can be adopted as strategies to increase the detection period of neutralizing antibodies in sera from these animals. In addition, further studies are needed to find ways to prolong the humoral immune response induced by these recombinant vaccines, considering the efficacy results already achieved with this technology in different species [3,15,18,19] and given the limitations of conventional production. Tests involving other adjuvant molecules besides the aluminum hydroxide used in most commercial and recombinant vaccines can improve and prolong the efficacy of these vaccines [21,28] and bioinformatic tools can be used for the development of new immunogenic recombinant molecules. Rodrigues et al. [29] reported that with the aid of such tools, they designed new versions of the rCPB protein and produced an unpurified protein (rCPB-C) with high productivity, solubility and antigenicity, which is another promising option for the development of recombinant vaccines that induce a more lasting humoral immune response. Probiotics can act as immunomodulators and enhancers of the immune response of animals after vaccination [30,31,32]. In a recent study, ewes immunized with a formulation containing the rETX protein of *C. perfringens*, after supplementation with *Bacillus toyonensis* BCT-7112T, showed a 2- to 3-fold increase in total serum levels of anti-rETX IgG compared to the group of no supplemented animals [33]. Thus, indicating that this practice can also be tested as one of the strategies to improve the immune response of cattle vaccinated with these recombinant trivalent *C. perfringens* toxoids. The present study showed the influence of antigen concentrations on the levels and duration of the humoral immune response against the main toxins of *C. perfringens* in cattle over a 12 month interval. It provides baseline data for future experiments aimed at obtaining adequate adjustments in the concentrations of these recombinant proteins as an attempt to prolong the protection period induced by this trivalent vaccine and for use in other species.

## 4. Conclusions

The 200 and 400 µg recombinant vaccines were the best formulations at inducing anti-CPA, anti-CPB and anti-ETX titers in cattle, with higher, more persistent titers detectable for a longer time than the commercial vaccine induced. From the standpoint of technology transfer to the animal vaccine production industry, the RV2 formulation (200 µg) would be the most economical, though both 400 and 200 µg can be considered safe and effective options for commercial production of clostridial vaccines, surpassing the limits of conventional production. However, none of the tested vaccine formulations stimulated the production of detectable neutralizing antibody titers for 1 year according to the serum neutralization test, which could suggest that the animals would not be protected if they were challenged by clostridial toxins after, for example, 180 days since vaccination. Therefore, these results reiterate what has long been discussed in preventive veterinary medicine. There is the need to reassess the current vaccination protocols and schedules, the need to adjust revaccination periods and the need to continuously conduct research on new vaccine molecules and adjuvants, including the use of probiotics, to promote a longer measurable humoral immune response of animals vaccinated against the alpha, beta and epsilon toxins of *Clostridium perfringens*.

## 5. Materials and Methods

### 5.1. Ethics Declaration

The study was conducted according to the norms regulated by the National Council for Animal Experimentation Control (Conselho Nacional de Controle de Experimentação Animal—CONCEA) and approved by the Ethics Committee on Animal Use of the Federal University of Pará (Universidade Federal do Pará—UFPA) under registration number 2448250321, date of approval 24 March 2020.

### 5.2. Recombinant Vaccines

The rCPA, rCPB and rETX proteins were produced as described by Moreira et al. [3]. Briefly, the recombinant plasmids expression vector were transformed into *E. coli* BL21 (DE3) strain pLysS by heat-shock. The transformed bacteria were grown in 50 mL Luria-Bertani LB containing ampicillin (100 g/mL) and chloramphenicol (25 μg/mL) at 37 °C for 16 h. Then, this volume was transferred to 500mL of the same medium and induced with IPTG to a final concentration of 0.5 mM for 5 h under the same conditions when culture reached OD600 = 0.5–0.8. The fractions containing the expressed proteins were verified by anti-His Western blot, purified by Ni-affinity chromatography and quantified by BCA protein assay kit (Thermo Scientific, Waltham, MA, USA). Purified rCPA, rCPB and rETX were lyophilized until the use. In the formulation of the recombinant trivalent vaccines, the proteins produced were made at concentrations of 100 μg (recombinant vaccine 1—RV1), 200 μg (recombinant vaccine 2—RV2) and 400 μg (recombinant vaccine 3—RV3), adsorbed to aluminum hydroxide [2.5–3.5% Al (OH)_3_], and kept under slight stirring for 20 h at 25 °C [34]. At the end of the production process, three recombinant trivalent vaccine formulations were obtained, containing, respectively, 100 (RV1), 200 (RV2) and 400 µg (RV3) of each of the recombinant proteins, rCPA, rCPB and rETX per vaccine dose.

### 5.3. Sterility and Safety Test

The recombinant vaccine sterility test was performed as described in norm 49 of the Brazilian Ministry of Agriculture, Livestock, and Supply (MAPA, for its name in Portuguese) [23]. In the safety test [18], two cattle were inoculated with a dose of the vaccine formulation containing 800 µg of each recombinant protein, double the dose of the vaccine with the highest concentration tested in the experiment (400 µg). These animals were observed daily for 7 days for the occurrence of local and systemic effects.

### 5.4. Vaccination of Animals

A total of 40 18-month-old Nelore cattle were used, which were kept in pasture with free access to water and mineral supplementation. These animals did not have detectable levels of antibodies against CPA, CPB or ETX according to the serum neutralization technique performed before the beginning of the experiment and were randomly allocated into five groups (G1–G5), each containing eight animals. The animals from G1, G2 and G3 received recombinant vaccines RV1, RV2 and RV3 (concentrations of 100, 200 and 400 µg), respectively, in a total volume of 2 mL per dose. The animals from G4 (positive control) were immunized with 2 mL per dose of the commercial vaccine (COMV) containing aluminum hydroxide as an adjuvant and the CPA, CPB and ETX toxoids of *C. perfringens*, among other clostridial toxoids. The animals from G5 (negative control) were inoculated with 2 mL of sterile saline solution (0.9% NaCl). The shots were given subcutaneously in the neck area on days 0 and 28. Blood samples were collected by jugular venipuncture on days 0, 56, 90, 120, 150, 180, 210, 240, 270, 300, 330 and 365. After collection, the samples were centrifuged in the laboratory (3000× *g*, 7 min), and the sera were labeled and stored at −20 °C until titration.

### 5.5. Evaluation of the Humoral Immune Response

Individual sera were titrated using the serum neutralization technique following the methods established by the United States Department of Agriculture [35] for detection of the alpha antitoxin (anti-CPA) and the methods described by European Pharmacopoeia (1998) [36] for the beta (anti-CPB) and epsilon antitoxins (anti-EXT). The antibody titer was calculated by the Reed and Muench method [37] and expressed in international units per milliliter (IU/mL).

### 5.6. Statistical Analysis

To evaluate the effects of vaccine type on the antibody titer, the Kruskal–Wallis nonparametric statistical test with repeated measures was used because the tested data did not meet the assumptions of a parametric test. Dunn’s post hoc test was applied to significant results. The calculations were completed in R 4.1 (R Development Core Team 2019). Descriptive statistical analysis was used to evaluate the antibody titer production curve for each vaccine tested over time *p*-values ≤ 0.05 were considered significant.

## Figures and Tables

**Table 1:** Main diseases caused by Clostridium perfringens toxinotypes in ruminant.

Toxinotype	Toxins	Diseases (Affected Animals)
A	CPA	Gas gangrene (all ruminants) and enterotoxemia (ovine)
	CPA, CPE	Enteritis (caprine)
	CPA, CPB2	Abomasitis (calves)
B	CPA, CPB, ETX	Necrotic enteritis and hemorrhagic enterotoxemia (bovine and ovine)
C	CPA, CPB	Necrotic enteritis and enterotoxemia (bovine, ovine and caprine)
D	CPA, ETX	Enterotoxemia (ovine, bovine, and caprine)
E	CPA, ITX	Hemorrhagic enteritis (lambs, and calves)

Adapted from Ferreira et al. [2].

**Table 2:** Percentage of animals immunized with the commercial (COMV) and recombinant trivalent vaccines (RV1, RV2, RV3) that showed the minimum antibody titers required by law for antitoxin alpha (anti-CPA), beta (anti-CPB) and epsilon (anti-ETX) at 56 days after the first vaccination.

	^a^ Seroconversion Rate	Anti-CPA (56 Days)	Anti-CPB (56 Days)	Anti-ETX (56 Days)
Vaccines				
COMV		37.5%	75%	100%
RV1		37.5%	37.5%	62.5%
RV2		100%	100%	100%
RV3		100%	100%	100%

^a^ Seroconversion rate according to the minimum neutralizing antibody values required by the Brazilian Ministry of Agriculture, Livestock, and Food Supply (MAPA).

## References

[1] ABIEC—Associação Brasileira Das Indústrias Exportadoras de Carnes. http://abiec.com.br/.

[2] Ferreira M., Moreira G., Cunha C., Mendonça M., Salvarani F., Moreira Â., Conceição F. (2016). Recombinant Alpha, Beta, and Epsilon Toxins of *Clostridium perfringens*: Production Strategies and Applications as Veterinary Vaccines. Toxins.

[3] Moreira G.M.S.G., Salvarani F.M., da Cunha C.E.P., Mendonça M., Moreira Â.N., Gonçalves L.A., Pires P.S., Lobato F.C.F., Conceição F.R. (2016). Immunogenicity of a Trivalent Recombinant Vaccine Against *Clostridium perfringens* Alpha, Beta, and Epsilon Toxins in Farm Ruminants. Sci. Rep..

[4] Silva R.O.S., Duarte M.C., Oliveira Junior C.A., de Assis R.A., Lana A.M.Q., Lobato F.C.F. (2018). Comparison of Humoral Neutralizing Antibody Response in Rabbits, Guinea Pigs, and Cattle Vaccinated with Epsilon and Beta Toxoids from *Clostridium perfringen*s and C. Botulinum Types C and D Toxoids. Anaerobe.

[5] Uzal F.A., Vidal J.E., McClane B.A., Gurjar A.A. (2010). Clostridium perfringens Toxins Involved in Mammalian Veterinary Diseases. Open Toxinol. J..

[6] Rood J.I., Adams V., Lacey J., Lyras D., McClane B.A., Melville S.B., Moore R.J., Popoff M.R., Sarker M.R., Songer J.G. (2018). Expansion of the *Clostridium perfringens* Toxin-Based Typing Scheme. Anaerobe.

[7] Abdel-Glil M.Y., Thomas P., Linde J., Busch A., Wieler L.H., Neubauer H., Seyboldt C. (2021). Comparative in Silico Genome Analysis of *Clostridium perfringens* Unravels Stable Phylogroups with Different Genome Characteristics and Pathogenic Potential. Sci. Rep..

[8] Mehdizadeh Gohari I., Navarro M.A., Li J., Shrestha A., Uzal F., McClane B.A. (2021). Pathogenicity and Virulence of *Clostridium perfringens*. Virulence.

[9] Geier R.R., Rehberger T.G., Smith A.H. (2021). Comparative Genomics of *Clostridium perfringens* Reveals Patterns of Host-Associated Phylogenetic Clades and Virulence Factors. Front. Microbiol..

[10] Lobato F.C.F., Lima C.G.R.D., Assis R.A., Pires P.S., Silva R.O.S., Salvarani F.M., Carmo A.O., Contigli C., Kalapothakis E. (2010). Potency against Enterotoxemia of a Recombinant *Clostridium perfringens* Type D Epsilon Toxoid in Ruminants. Vaccine.

[11] Ferreira M.R.A., dos Santos F.D., da Cunha C.E.P., Moreira C., Donassolo R.A., Magalhães C.G., Belo Reis A.S., Oliveira C.M.C., Barbosa J.D., Leite F.P.L. (2018). Immunogenicity of *Clostridium perfringens* Epsilon Toxin Recombinant Bacterin in Rabbit and Ruminants. Vaccine.

[12] Moreira C., Ferreira M., da Cunha C., Donassolo R., Finger P., Moreira G., Otaka D., de Sousa L., Barbosa J., Moreira Â. (2018). Immunogenicity of a Bivalent Non-Purified Recombinant Vaccine against Botulism in Cattle. Toxins.

[13] Gonçalves L.A., Lobato Z.I.P., Silva R.O.S., Salvarani F.M., Pires P.S., Assis R.A., Lobato F.C.F. (2009). Selection of a *Clostridium perfringens* Type D Epsilon Toxin Producer via Dot-Blot Test. Arch. Microbiol..

[14] Titball R.W. (2009). *Clostridium perfringens* Vaccines. Vaccine.

[15] Salvarani F.M., Conceição F.R., Cunha C.E.P., Moreira G.M.S.G., Pires P.S., Silva R.O.S., Alves G.G., Lobato F.C.F. (2013). Vaccination with Recombinant *Clostridium perfringens* Toxoids α and β Promotes Elevated Antepartum and Passive Humoral Immunity in Swine. Vaccine.

[16] Ferreira M.R.A., Motta J.F., Azevedo M.L., dos Santos L.M., Júnior C.M., Rodrigues R.R., Donassolo R.A., dos Reis A.S.B., Barbosa J.D., Salvarani F.M. (2019). Inactivated Recombinant *Escherichia coli* as a Candidate Vaccine against *Clostridium perfringens* Alpha Toxin in Sheep. Anaerobe.

[17] Otaka D., Barbosa J., Moreira C., Ferreira M., Cunha C., Brito A., Donassolo R., Moreira Â., Conceição F., Salvarani F. (2017). Humoral Response of Buffaloes to a Recombinant Vaccine against Botulism Serotypes C and D. Toxins.

[18] Otaka D.Y., Barbosa J.D., de Souza L.A., Moreira C., Ferreira M.R.A., Donassolo R.A., Conceição F.R., Salvarani F.M. (2020). Recombinant Vaccine against Botulism in Buffaloes: Evaluation of the Humoral Immune Response over 12 Months. Anaerobe.

[19] Freitas N.F.Q.R., Barbosa J.D., Otaka D.Y., Ferreira M.R.A., Rodrigues R.R., Moreira C., Conceição F.R., Salvarani F.M. (2020). *Clostridium perfringens* α and β Recombinant Toxoids in Equine Immunization. Pesq. Vet. Bras..

[20] Moreira C., Ferreira M.R.A., Finger P.F., Magalhães C.G., Cunha C.E.P., Rodrigues R.R., Otaka D.Y., Galvão C.C., Salvarani F.M., Moreira Â.N. (2020). Protective Efficacy of Recombinant Bacterin Vaccine against Botulism in Cattle. Vaccine.

[21] Jiang Z., De Y., Chang J., Wang F., Yu L. (2014). Induction of Potential Protective Immunity against Enterotoxemia in Calves by Single or Multiple Recombinant *Clostridium perfringens* Toxoids: Immunogenicity of Recombinant Toxoids. Microbiol. Immunol..

[22] Augusto de Oliveira C., Duarte M.C., Antunes de Assis R., Alves G.G., Silva R.O.S., Faria Lobato F.C. (2019). Humoral Responses in Cattle to Commercial Vaccines Containing *Clostridium perfringen*s Epsilon Toxoid and *C. botulinum* Types C and D Toxoids Last Less than a-Year. Anaerobe.

[23] Ministério Da Agricultura Pecuária e Abastecimento Do Brasil (MAPA) Portaria n 49;1997. http://sistemasweb.agricultura.gov.br/sislegis/action/detalhaAto.do?method=visualizarAtoPortalMapa&chave=1924117692.

[24] Rossi A., Mónaco A., Guarnaschelli J., Silveira F., Iriarte A., Benecke A.G., Chabalgoity J.A. (2018). Temporal Evolution of Anti- Clostridium Antibody Responses in Sheep after Vaccination with Polyvalent Clostridial Vaccines. Vet. Immunol. Immunopathol..

[25] Schwartzkoff C., Egerton J., Stewart D., Lehrbach P., Elleman T., Hoyne P. (1993). The Effects of Antigenic Competition on the Efficacy of Multivalent Footrot Vaccines. Aust. Veter. J..

[26] Hunt J.D., Jackson D.C., Brown L.E., Wood P.R., Stewart D.J. (1994). Antigenic Competition in a Multivalent Foot Rot Vaccine. Vaccine.

[27] Simpson K.M., Callan R.J., Van Metre D.C. (2018). Clostridial Abomasitis and Enteritis in Ruminants. Vet. Clin. N. Am. Food Anim. Pract..

[28] Jang S.I., Lillehoj H.S., Lee S.-H., Lee K.W., Lillehoj E.P., Hong Y.H., An D.-J., Jeong W., Chun J.-E., Bertrand F. (2012). Vaccination with *Clostridium perfringens* Recombinant Proteins in Combination with Montanide^TM^ ISA 71 VG Adjuvant Increases Protection against Experimental Necrotic Enteritis in Commercial Broiler Chickens. Vaccine.

[29] Rodrigues R.R., Alves Ferreira M.R., Donassolo R.A., Ferreira Alves M.L., Motta J.F., Junior C.M., Salvarani F.M., Moreira A.N., Conceicao F.R. (2021). Evaluation of the Expression and Immunogenicity of Four Versions of Recombinant *Clostridium perfringens* Beta Toxin Designed by Bioinformatics Tools. Anaerobe.

[30] Roos T.B., de Moraes C.M., Sturbelle R.T., Dummer L.A., Fischer G., Leite F.P.L. (2018). Probiotics Bacillus Toyonensis and Saccharomyces Boulardii Improve the Vaccine Immune Response to Bovine Herpesvirus Type 5 in Sheep. Res. Vet. Sci..

[31] Roos T.B., Tabeleão V.C., Dümmer L.A., Schwegler E., Goulart M.A., Moura S.V., Corrêa M.N., Leite F.P.L., Gil-Turnes C. (2010). Effect of *Bacillus cereus* Var. Toyoi and *Saccharomyces boulardii* on the Immune Response of Sheep to Vaccines. Food Agric. Immunol..

[32] Santos F.D.S., Mazzoli A., Maia A.R., Saggese A., Isticato R., Leite F., Iossa S., Ricca E., Baccigalupi L. (2020). A Probiotic Treatment Increases the Immune Response Induced by the Nasal Delivery of Spore-Adsorbed TTFC. Microb. Cell Fact..

[33] Santos F.D.S., Ferreira M.R.A., Maubrigades L.R., Gonçalves V.S., Lara A.P.S., Moreira C., Salvarani F.M., Conceição F.R., Leivas Leite F.P. (2021). *Bacillus toyonensis* BCT-7112 ^T^ Transient Supplementation Improves Vaccine Efficacy in Ewes Vaccinated against *Clostridium perfringens* Epsilon Toxin. J. Appl. Microbiol..

[34] Souza A.M., Reis J.K.P., Assis R.A., Horta C.C., Siqueira F.F., Facchin S., Alvarenga E.R., Castro C.S., Salvarani F.M., Silva R.O.S. (2010). Molecular Cloning and Expression of Epsilon Toxin from *Clostridium perfringens* Type D and Tests of Animal Immunization. Genet. Mol. Res..

[35] United States Department of Agriculture (USDA) (2002). Conditional Licenses for Products Containing Clostridium Perfringens Type A.

[36] COE (Council of Europe) (1998). European Pharmacopoeia.

[37] Reed L.J., Muench H. (1938). A simple method of estimating fifty per cent endpoints l’2. Am. J. Epidemiol..

